# Supplementary material for: The genome sequence of Frangula alnus Mill., 1768 (Rosales: Rhamnaceae)
Source: Wellcome Open Res. 2026 Jul 30;11:492. [Version 1] doi: 10.12688/wellcomeopenres.27238.1 (PMC13562949; doi:10.12688/wellcomeopenres.27238.1)
Supplement: Peer review history

# Reviewer report: Reviewer response for version 1

**DOI:** 10.21956/wellcomeopenres.29979.r163251

**Published:** 2026-09-04

**Authors:** Hari Paul Sankhyan, Sumit Nangla

**Affiliations:** 1 Dr. Yashwant Singh Parmar University of Horticulture and Forestry, Solan, India; 2 Tree Improvement and Genetic Resources, Dr Yashwant Singh Parmar University of Horticulture and Forestry, Nauni-Solan (H.P.), Nauni, Solan, Himachal Pradesh, India

Summary

 The manuscript presents a high-quality, chromosome-level genome assembly of *Frangula alnus* (alder buckthorn), generated as part of the Darwin Tree of Life project. The authors provide a phased genome assembly comprising two haplotypes, together with mitochondrial and plastid genome sequences. The study is supported by extensive specimen documentation, flow cytometry, DNA barcoding, PacBio HiFi sequencing, Hi-C scaffolding, and a well-described assembly and quality-assessment workflow.

 The manuscript provides a clear biological and scientific rationale for generating a reference genome for *F. alnus*. The species is ecologically important, including its role as a host plant for specialist species such as the Brimstone butterfly, and genomic resources may contribute to future studies on adaptation, environmental resilience, and coevolutionary relationships. The assembly therefore represents a valuable genomic resource for plant evolutionary biology, ecology, conservation, and comparative genomics.

 Overall, the manuscript is well structured, technically sound, and provides a valuable genomic dataset. The genome assembly appears to be of high quality, with most of both haplotypes assigned to chromosomal pseudomolecules and supported by multiple complementary quality assessments.

 Assessment of the specific questions

**1. Is the rationale for creating the dataset(s) clearly described? — Yes**

 The rationale for generating the *Frangula alnus* genome assembly is clearly explained. The manuscript provides sufficient biological and ecological background on the species and identifies the potential value of a high-quality genome sequence for investigating adaptation, resilience, genetic variation, and ecological interactions. The inclusion of this species within the Darwin Tree of Life initiative further provides a clear broader rationale for the generation of the dataset.

**2. Are the protocols appropriate and is the work technically sound? — Yes**

 The experimental and computational approaches are appropriate for the generation of a high-quality plant reference genome. The use of PacBio HiFi sequencing in combination with Hi-C data and haplotype-aware assembly provides a robust strategy for producing phased, chromosome-level assemblies.

 The workflow includes appropriate specimen identification and validation, DNA barcoding, high molecular weight DNA extraction, long-read sequencing, Hi-C library preparation, genome assembly, scaffolding, and quality assessment. The use of complementary assembly evaluation approaches, including *k*-mer analysis, Merqury-based quality assessment, BUSCO completeness assessment, and BlobToolKit visualisation, provides substantial support for the quality and completeness of the final assembly.

 The reported quality metrics are also convincing. The manuscript reports high estimated consensus quality values and high combined *k*-mer completeness, while BUSCO analysis recovered 98.2% of the expected eudicot gene set for haplotype 1. The assembly therefore meets a high standard for a reference genome resource.

**3. Are sufficient details of methods and materials provided to allow replication by others? — Yes**

 The manuscript provides substantial methodological detail covering specimen collection, taxonomic identification, herbarium voucher deposition, genome size estimation, DNA barcoding, DNA extraction, library preparation, sequencing, Hi-C generation, genome assembly, scaffolding, and quality assessment.

 The authors also provide references to established protocols and standard operating procedures, including protocols for DNA extraction, sample processing, sequencing, and genome analysis. Software tools and versions are reported, which further improves reproducibility.

 The description of the sequencing and assembly workflow is sufficiently detailed to enable researchers to understand and reproduce the general analytical approach, particularly when used in conjunction with the cited protocols and software documentation.

**4. Are the datasets clearly presented in a useable and accessible format? — Yes**

 The datasets are clearly presented through tables, figures, accession information, and genome assembly statistics. The manuscript provides information on the specimen and sequencing dataset, assembly statistics, chromosomal pseudomolecules, organellar genome assemblies, and assembly quality metrics.

 The presentation of the two haplotypes is particularly useful, and the use of Hi-C contact maps, *k*-mer spectra, Merqury assessment, BUSCO analysis, and BlobToolKit plots provides multiple complementary perspectives on assembly quality. The manuscript also clearly reports regions where scaffold order and orientation remain uncertain, which is an important and transparent aspect of genome resource reporting.

 Overall assessment

 This manuscript presents a valuable and high-quality genomic resource for *Frangula alnus*. The rationale for producing the dataset is clearly articulated, the experimental and computational protocols are appropriate and technically sound, and the methods are described with sufficient detail to support reproducibility. The genome assembly is supported by comprehensive quality-control analyses and appears to meet a high standard for a chromosome-level reference genome.

 The manuscript is clearly written and appropriately structured for a genome resource publication. I did not identify any major scientific concerns that would compromise the validity or utility of the dataset.

 Recommendation

**All four assessment questions: Yes.**

 In my opinion, the manuscript is scientifically sound and the dataset represents a useful contribution to genomic resources for *Frangula alnus* and related research areas. I recommend indexing in its present form, subject to any routine editorial corrections.

Are sufficient details of methods and materials provided to allow replication by others?

Yes

Is the rationale for creating the dataset(s) clearly described?

No

Are the datasets clearly presented in a useable and accessible format?

Yes

Are the protocols appropriate and is the work technically sound?

Yes

Reviewer Expertise:

Forestry; Plant Genetics and Breeding; Tree Improvement; Plant Genomics; Forest Biotechnology; Biodiversity and Conservation Genetics

We confirm that we have read this submission and believe that we have an appropriate level of expertise to confirm that it is of an acceptable scientific standard.

# Reviewer report: Reviewer response for version 1

**DOI:** 10.21956/wellcomeopenres.29979.r163249

**Published:** 2026-08-06

**Authors:** S Brooks Parrish

**Affiliations:** 1 University of Florida, Gainesville, Florida, USA

This manuscript presents a high-quality, chromosome-scale, haplotype-resolved genome assembly for *Frangula alnus*, alder buckthorn. The rationale for generating this dataset is clearly described. *F. alnus* is an ecologically important and conservation-relevant species, including its role as a host plant for the Common Brimstone butterfly, and the genome will support future studies of adaptation, resilience, and species interactions. The assembly consists of two haplotypes of 279.06 Mb and 281.25 Mb, with 99.91% of each haplotype scaffolded into 10 chromosomal pseudomolecules. Mitochondrial and plastid assemblies are also reported.

 The protocols are appropriate and the work is technically sound. The authors used suitable procedures for sample collection, species verification by DNA barcoding, genome size estimation by flow cytometry and k-mer analysis, PacBio HiFi sequencing, Hi-C scaffolding, assembly, manual curation, and quality assessment. The reported metrics indicate an excellent assembly, including scaffold N50 values of 24.62 Mb and 25.06 Mb, high consensus quality values, combined k-mer completeness of 99.03%, and BUSCO completeness of 98.2%. Sufficient methodological detail, software versions, specimen information, and accession numbers are provided to support reproducibility.

 The datasets are clearly presented in usable and accessible formats, with raw sequence data and assemblies deposited in INSDC databases. I have no major concerns and no points that must be addressed to make the article scientifically sound. I recommend approval. As minor editorial suggestions, the authors may want to clarify the difference between the flow-cytometry genome size estimate and final assembly, comment on the practical implications of the noted uncertain chromosome regions and chromosome 8 inversion, and correct the typo “Alder Bbckthorn.”

Are sufficient details of methods and materials provided to allow replication by others?

Yes

Is the rationale for creating the dataset(s) clearly described?

Yes

Are the datasets clearly presented in a useable and accessible format?

Yes

Are the protocols appropriate and is the work technically sound?

Yes

Reviewer Expertise:

Plant Breeding, Bioinformatics, Molecular Biology

I confirm that I have read this submission and believe that I have an appropriate level of expertise to confirm that it is of an acceptable scientific standard.
